# Supplementary material for: Protein determinants of dissemination and host specificity of metallo-β-lactamases
Source: Nat Commun. 2019 Aug 9;10:3617. doi: 10.1038/s41467-019-11615-w (PMC6689000; doi:10.1038/s41467-019-11615-w)
Supplement: Supplementary file 1 — Supplementary Information [file 41467_2019_11615_MOESM1_ESM.pdf]

Supplementary Information

Protein determinants of dissemination and host specificity of Metallo-β-lactamases

López et al.

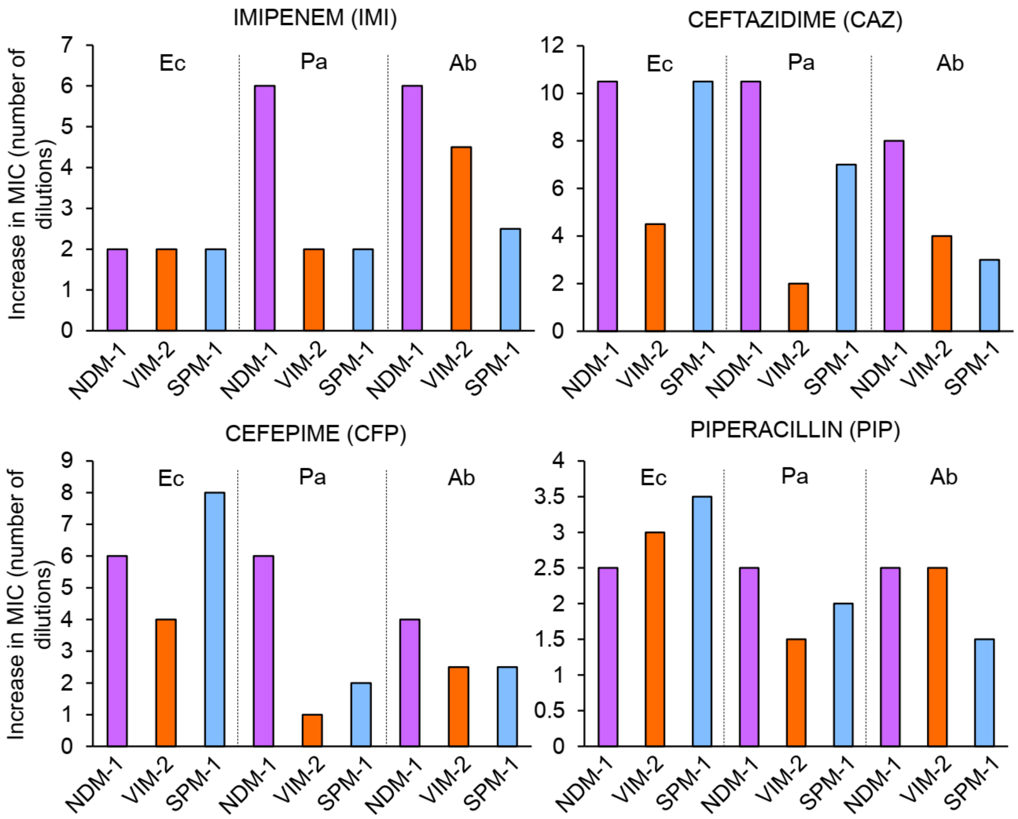

Supplementary Figure 1│The resistance phenotype of MBLs in different hosts does not correlate with bacterial host preferences. Relative MIC values (shown as number of dilutions over control strain: cells harboring an empty vector) of imipenem (IMI), ceftazidime (CAZ), cefepime (CFP) and piperacillin (PIP) for *E. coli*, *P. aeruginosa* and *A. baumannii* expressing *bla*$_{\text{NDM-1}}$, *bla*$_{\text{VIM-2}}$, *bla*$_{\text{SPM-1}}$ at 10 µM of IPTG. Data correspond to mean values from three independent experiments. Source data are provided as a Source Data file.

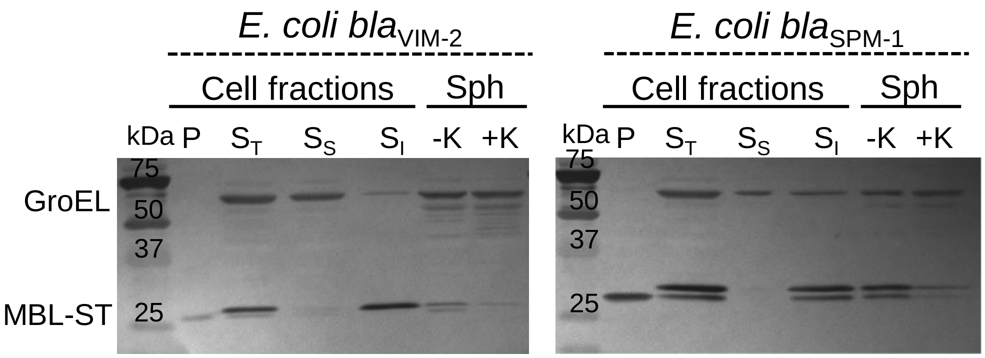

Supplementary Figure 2│ Localization of precursor forms of VIM-2 and SPM-1 in *E. coli* cells. Immunoblot of periplasmic (P) and spheroplast (S_T) fractions of *E. coli* cells expressing VIM-2 or SPM-1. Cells were fractioned by EDTA-Lysozyme treatment, obtaining spheroplasts (Sph) which were lysed and separated into soluble (S_S) and insoluble (S_I) fractions, or treated with (+K) or without (-K) Proteinase K. GroEL was used as a cytoplasmic marker. This panel is representative of two independent experiments. Source data are provided as a Source Data file.

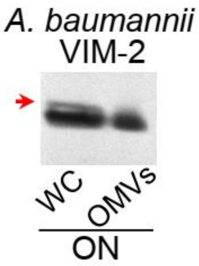

Supplementary Figure 3│ Accumulation of the precursor forms from *A. baumannii* expressing VIM-2. VIM-2 levels (fused to a C-terminal ST) by immunoblot analysis, using anti-Strep tag antibodies, in whole cells (WC) and in OMVs purified from its cell culture supernatants from *A. baumannii* expressing $bla_{\text{VIM-2}}$, after overnight induction at 20 µM with IPTG. The red arrow indicates the precursor form. This panel is representative of two independent experiments. Source data are provided as a Source Data file.

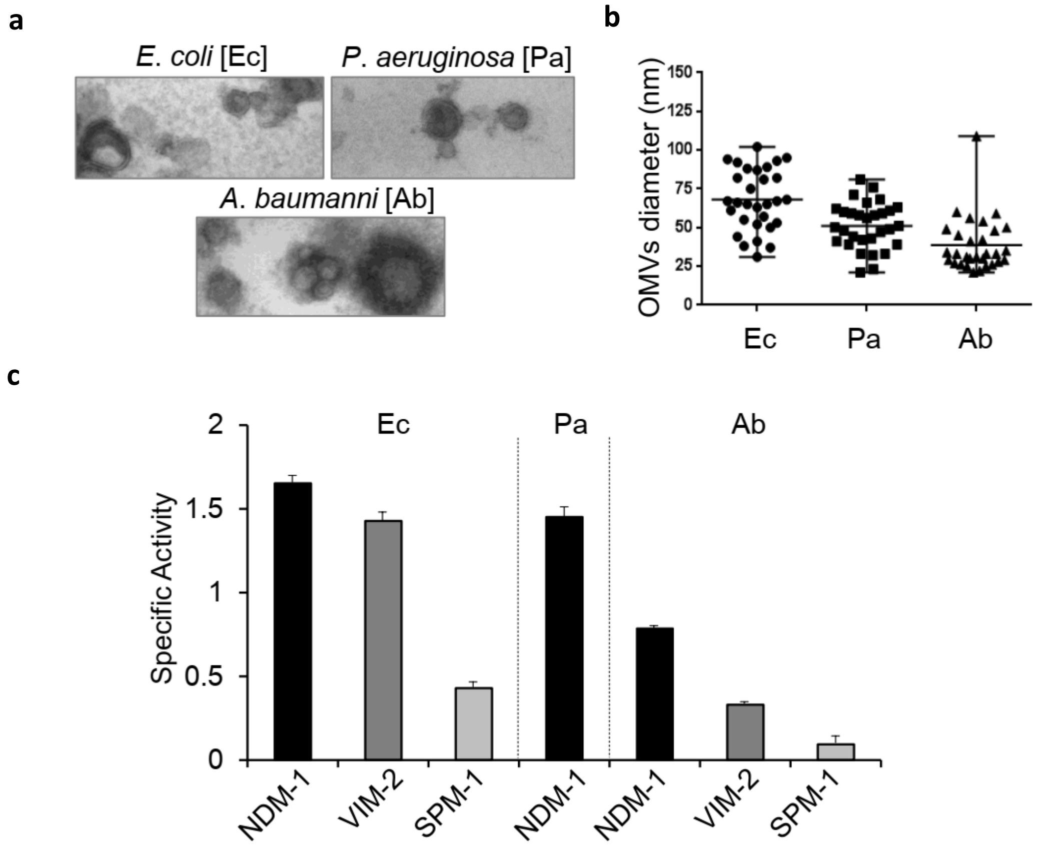

Supplementary Figure 4│ Analysis of the OMVs. TEM showed the presence of homogeneous and spherical vesicles free of contaminants. (**a**) Negative staining transmission electron microscopy (TEM) of OMVs from *E. coli*, *P. aeruginosa* and *A. baumannii* cells expressing $bla_{\text{NDM-1}}$. (**b**) Diameter ranges expressed in nm of OMVs from *E. coli*, *P. aeruginosa* and *A. baumannii* cells expressing $bla_{\text{NDM-1}}$. (**c**) Specific activities of each MBL, determined as the hydrolysis of imipenem per the protein of the MBL (according to the quantification by immunoblot, Fig. 1a), in the OMVs purified form different bacterial hosts. Source data are provided as a Source Data file.

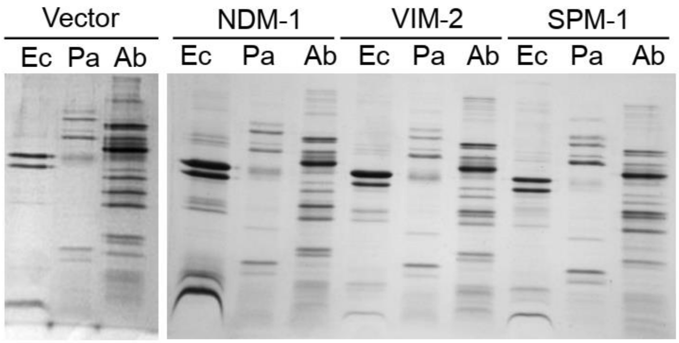

Supplementary Figure 5│ MBLs expression does not affect the overall protein profiles in OMVs. OMVs purified from *E*. *coli*, *P*. *aeruginosa* and *A*. *baumannii* carrying the empty vector (Vector) or expressing $bla_{\text{NDM-1}}$, $bla_{\text{VIM-2}}$ or $bla_{\text{SPM-1}}$ were subjected to ultracentrifugation in a sucrose solution and analyzed by SDS-PAGE. This panel is representative of two independent experiments. Source data are provided as a Source Data file.

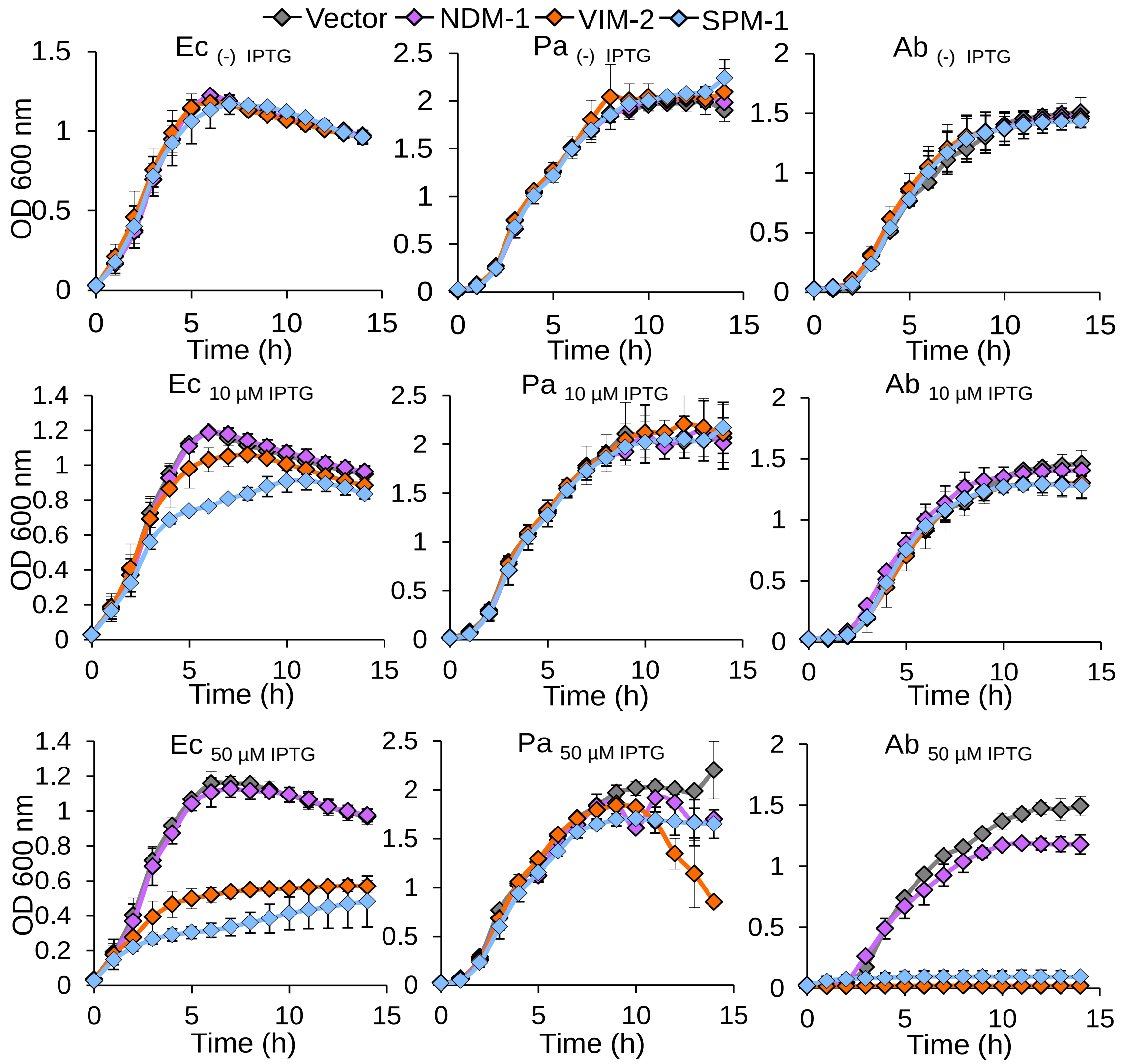

Supplementary Figure 6│ Higher expression levels of VIM-2 and SPM-1, in non-frequent hosts, compromises to a greater extent the fitness. Growth curves for *E. coli*, *P. aeruginosa* and *A. baumannii* carrying empty vector (Vector) or expressing *bla*$_{\text{NDM-1}}$, *bla*$_{\text{VIM-2}}$ or *bla*$_{\text{SPM-1}}$ in aerobic condition in LB medium at 37°C without (-) or with increasing IPTG concentration (10 or 50 µM of IPTG), as indicated. OD600 nm of the cultures was recorded every hour for 15 h. Data represent the mean (± s.d) of three independent experiments. Source data are provided as a Source Data file.

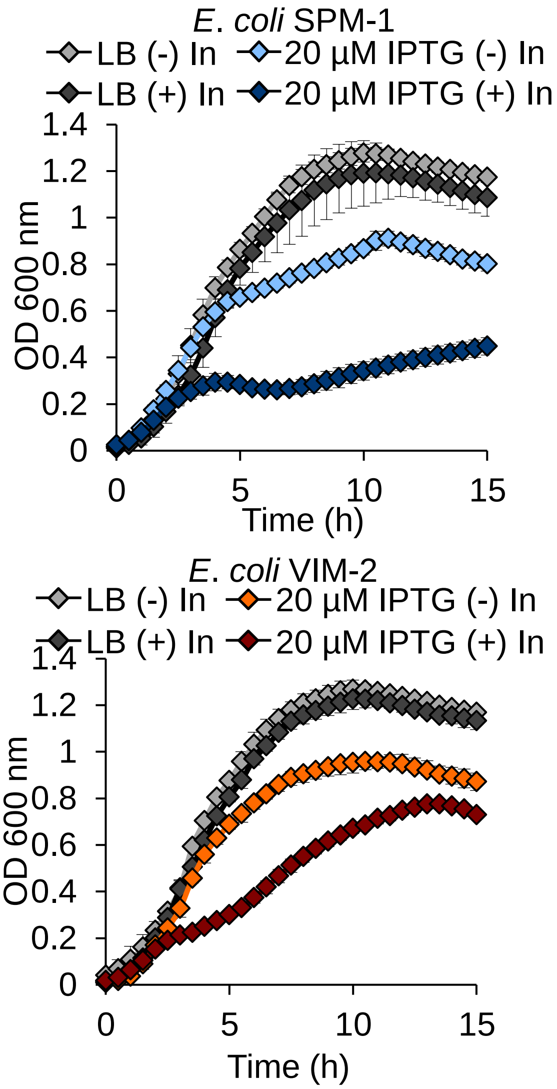

Supplementary Figure 7| Impact of the enzymatic activity on bacterial fitness. Inhibition of carbapenemase activity does not rescue growth reduction observed upon expression of *bla*$_{SPM-1}$ or *bla*$_{VIM-2}$. Growth curves of *E. coli* cells harboring *bla*$_{SPM-1}$ or *bla*$_{VIM-2}$ in aerobic conditions in LB medium at 37°C without or with the addition 20 µM of IPTG and with the addition of an enzymatic activity inhibitor (+In) or not (-In), as indicated. OD600nm of the cultures was recorded every half hour for 15 h. Data depict the mean value (± s.d) of three independent experiments. Source data are provided as a Source Data file.

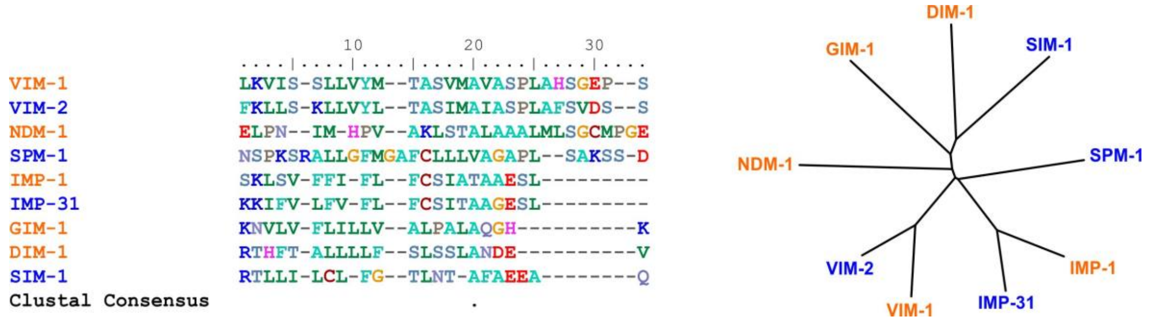

Supplementary Figure 8│ Relation between MBLs host range and signal peptide sequences. *Left panel*: multiple sequence alignment of broad host range IMP-1, NDM-1, GIM-1, DIM-1 and VIM-1 (orange), and narrow host range VIM-2, SPM-1, IMP-31 and SIM-1 (blue). *Right panel*: consensus phylogenetic tree calculated from multiple sequence alignment, with LG substitution model and 100 bootstraps, using the maximum likelihood algorithm PhyML.

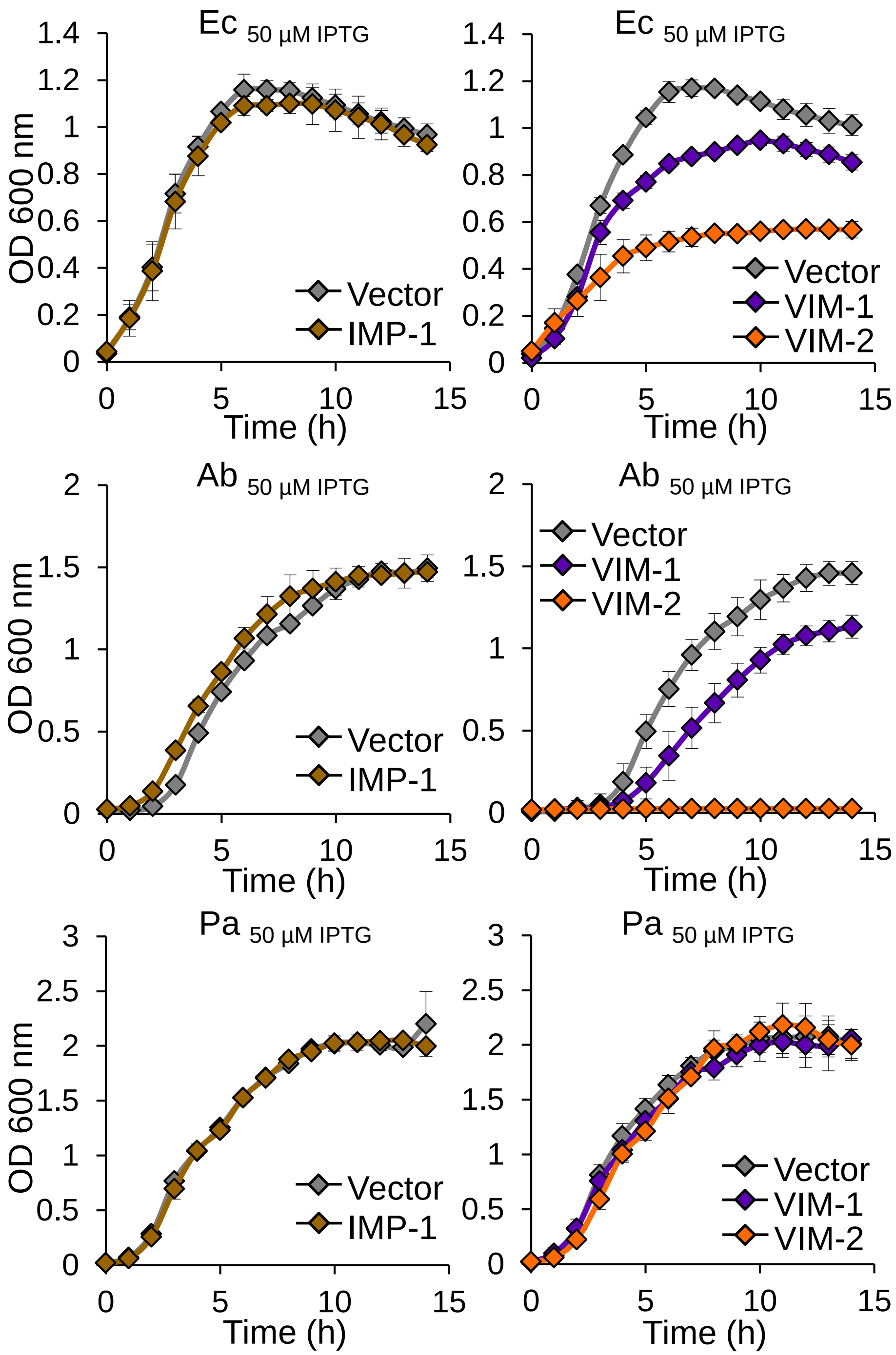

Supplementary Figure 9│ Expression of IMP-1 or VIM-1 does not compromise fitness of *E. coli, A. baumannii* and *P. aeruginosa*. Growth curves for *E. coli*, *P. aeruginosa* and *A. baumannii* carrying empty vector (Vector) or expressing *bla*$_{IMP-1}$, *bla*$_{VIM-2}$ or *bla*$_{VIM-1}$ in aerobic conditions in LB medium at 37°C with 50 µM of IPTG, as indicated. OD600 nm of the cultures was recorded every hour for 15 h. Data represent the mean value (± s.d) of three independent experiments. Source data are provided as a Source Data file.

Supplementary Table 1│ β-lactam antibiotics MIC values for *E. coli*, *P. aeruginosa* and *A. baumannii* expressing *bla*$_{NDM-1}$, *bla*$_{VIM-2}$ and *bla*$_{SPM-1}$ at 10 µM of IPTG

		**MIC (µg/mL)**		
		E. coli		
	**Imipenem**	**Ceftazidime**	**Cefepime**	**Piperacillin**
**Vector**	0.25	0.5	0.03	2
**NDM-1**	1	512-1024	2	8-16
**VIM-2**	1	8-16	0.5	16
**SPM-1**	1	512-1024	0.5	16-32
		P. aeruginosa		
	**Imipenem**	**Ceftazidime**	**Cefepime**	**Piperacillin**
**Vector**	1-2	0.5	0.5	1
**NDM-1**	64-128	512-1024	32	4-8
**VIM-2**	4-8	2	1	2-4
**SPM-1**	4-8	64	16-32	4
		A. baumannii		
	**Imipenem**	**Ceftazidime**	**Cefepime**	**Piperacillin**
**Vector**	0.125-0.025	2-4	2	2-4
**NDM-1**	8-16	512-1024	32	16
**VIM-2**	4	32-64	8-16	16
**SPM-1**	1	256-512	16-32	8

Values were determined by triplicate

Supplementary Table 2│ Expression of VIM-2 and SPM-1 in a non-frequent host correlates with increased vesiculation, without Lpp crosslinking changes in PG

	Avg molar ratio (mol%)			
	**Vector**	**NDM-1**	**VIM-2**	**SPM-1**
**Muropeptide**				
**M3L**	3.1 ± 0.1	3.0± 0.2	3.8 ± 0.2	2.42 ± 0.02
**D33DL**	1.8 ± 0.1	1.67 ± 0.09	1.65 ± 0.06	1.7 ± 0.1
**D43L**	4.26 ± 0.06	4.0 ± 0.2	3.5± 0.2	3.76 ± 0.08
**T443L**	1.06 ± 0.01	0.97 ± 0.01	1.28 ± 0.01	0.88 ± 0.04
**D33DLN**	> 0.1	> 0.1	> 0.1	> 0.1
**D43LN**	0.5± 0.1	0.70 ± 0.06	0.59 ± 0.06	0.5 ± 0.2
**T443LN**	> 0.1	> 0.1	> 0.1	> 0.1

The molar ratios of the different muropeptide structures were calculated for *E. coli* strains harboring empty vector (Vector) or expressing NDM-1, VIM-2 or SPM-1. Data are the average molar ratio (mol%) plus standard deviation of two biological replicas for each strain. Only Lpp-crosslinked muropeptides are shown: M3L (monomer, NAcGlc-NAcMur-LAla-DGlu-Dap-Lys-Arg), D33L (dimer, NAcGlc-NAcMur-LAla-DGlu-Dap-Lys-Arg/NAcGlc-NAcMur-LAla-DGlu-Dap),D43L (dimer, NAcGlc-NAcMur-LAla-DGlu-Dap-Lys-Arg/NAcGlc-NAcMur-LAla-DGlu-Dap-DAla), T443L (trimer, NAcGlc-NAcMur-LAla-DGlu-Dap-Lys-Arg/NAcGlc-NAcMur-LAla-DGlu-Dap-DAla/NAcGlc-NAcMur-LAla-DGlu-Dap-DAla) D33DLN, (anhydro-dimer, NAcGlc-NAcMur-LAla-DGlu-Dap-Lys-Arg/NAcGlc-NAcMurAhn-LAla-DGlu-Dap), D43LN (anhydro-dimer, NAcGlc-NAcMur-LAla-DGlu-Dap-Lys-Arg/NAcGlc-NAcMurAhn-LAla-DGlu-Dap-DAla) and T443LN (anhydro-trimer, NAcGlc-NAcMur-LAla-DGlu-Dap-Lys-Arg/NAcGlc-NAcMur-LAla-DGlu-Dap-DAla/NAcGlc-NAcMurAhn-LAla-DGlu-Dap-DAla).
